# Phenolic Rich Extract from* Clinacanthus nutans* Attenuates Hyperlipidemia-Associated Oxidative Stress in Rats

**DOI:** 10.1155/2016/4137908

**Published:** 2016-01-10

**Authors:** Nadarajan Sarega, Mustapha Umar Imam, Der-Jiun Ooi, Kim Wei Chan, Norhaizan Md Esa, Norhasnida Zawawi, Maznah Ismail

**Affiliations:** ^1^Laboratory of Molecular Biomedicine, Institute of Bioscience, Universiti Putra Malaysia, 43400 Serdang, Selangor, Malaysia; ^2^Department of Nutrition and Dietetics, Faculty of Medicine and Health Sciences, Universiti Putra Malaysia, 43400 Serdang, Selangor, Malaysia; ^3^Department of Food Science, Faculty of Food Science and Technology, Universiti Putra Malaysia, 43400 Serdang, Selangor, Malaysia

## Abstract

*Clinacanthus nutans* is used as traditional medicine in Asia but there are limited scientific studies to support its use. In this study, the stem and leaf of* C. nutans* were extracted using solvents of differing polarities, and their antioxidant capacities were determined using multiple antioxidant assays. The water and aqueous methanolic leaf extracts were further fractionated and their antioxidant capacities and phenolic compositions were tested. Furthermore, the efficacies of the water and aqueous methanolic leaf extracts were tested against hyperlipidemia-induced oxidative stress in rats. Serum and hepatic antioxidant and oxidative stress markers were tested after feeding the rats with high fat diet together with the extracts or simvastatin for 7 weeks. The results indicated that both leaf extracts attenuated oxidative stress through increasing serum antioxidant enzymes activity and upregulating the expression of hepatic antioxidant genes. Multiple phenolic compounds were detected in the extracts and fractions of* C. nutans*, although protocatechuic acid was one of the most abundant and may have contributed significantly towards the bioactivities of the extracts. However, synergistic effects of different phenolics may have contributed to the overall bioactivities.* C. nutans* can be a good source of functional ingredients for the management of oxidative stress-related diseases.

## 1. Introduction

Hypercholesterolemia is a lipoprotein metabolic disorder characterized by altered metabolism of cholesterol, which promotes the production of reactive oxygen species (ROS) through modulation of the activities of enzymes like NADPH oxidase and xanthine oxidase. The altered activities of these enzymes have been demonstrated to disturb the endothelial superoxide anion production resulting in c-Jun-N-terminal kinase-mediated inactivation of endothelium-derived nitric oxide and subsequent increases in oxygen radical production and inflammation [1–3]. Conversely, oxidative stress due to an imbalance between the generation of ROS and the endogenous antioxidant systems has been reported to lead to increased lipid peroxidation, which in turn is involved in the etiology of several chronic diseases such as cardiovascular diseases (CVD), diabetes, obesity, and cancer [4].

Antioxidants attenuate or inhibit the oxidation of lipids or other biomolecules and thus prevent or repair the damage to body cells due to oxidation by free radical species [5]. Free radical species can be neutralized through dismutation or reduction by endogenous antioxidants like superoxide dismutase (SOD) and catalase, respectively, and through direct scavenging or electron transfer by exogenous antioxidants like vitamins C and E, respectively [6]. Additionally, plant bioactive compounds including polyphenols and flavonoids have been demonstrated to attenuate oxidative stress through multiple mechanisms including the regulation of free radical-induced oxidation of biomolecules [7]. Moreover, these plants have received heightened attention due to their perceived cost-effectiveness and lesser side effects compared to synthetic pharmaceutical agents [8]. Additionally, the presence of phenolic compounds correlates with the antioxidant activities of plants and thus there have been suggestions that antioxidant tests can be used as the major determinants for evaluating the antioxidant potentials of herbs [9]. Phenolic compounds are also known for their wide range of physiological properties including cardioprotection, anticancer, and neuroprotection [7].

*Clinacanthus nutans* belongs to the family of Acanthaceae and is widely used in Thailand and Indonesia as traditional medicine. In fact, it is categorized as an essential medicinal plant for primary health care of the Thai Ministry of Public Health [10]. This plant has been traditionally used in Asia to treat oxidative stress-related diseases such as CVD, diabetes, and various kinds of cancers. It is not surprising, therefore, that in recent years its extracts have been demonstrated to have blood glucose-lowering [11], antioxidant [12, 13], antiproliferative [13], and anti-inflammatory effects [14]. Numerous bioactives have been demonstrated in* C. nutans*, including phenolics, flavonoids, *β*-sitosterol, stigmasterol, and chlorophyll derivatives [15]. However, an extensive investigation of its phenolic compounds and their correlation with the antioxidative effects of this plant has not been demonstrated. This is important since the plant has potent antioxidative potentials likely mediated by its phenolics [12, 13]. Furthermore, the preparation of bioactive-rich fractions, having a lead compound and lesser amounts of other bioactive compounds, has conveniently gained momentum due to possible synergistic actions of different bioactives compared with the effects of individual bioactive compounds [16].

Thus, we prepared protocatechuic acid- (PCA-) rich fractions from* C. nutans*, followed by the determinations of their antioxidant activities and compositional analyses. We also evaluated their effects against oxidative stress in hypercholesterolemic rats. The findings could pave the way for development of functional ingredients from* C. nutans* for management of oxidative stress associated with hypercholesterolemia.

## 2. Materials and Methods

### 2.1. Reagents and Chemicals

Chemicals used for extraction including hexane, methanol, and ethyl acetate were of analytical grade and purchased from Thermo Fisher Scientific (Massachusetts, USA). The compounds, 2,2-diphenyl-2-picrylhydrazyl (DPPH), Folin-Ciocalteu's phenol reagent, potassium ferricyanide (K_3_[Fe(CN)_6_]), sodium dodecyl sulphate (SDS), ferric chloride (FeCl_3_), sodium carbonate (Na_2_CO_3_), 2,2′-azinothiazoline-6-sulphonic acid (ABTS), and potassium persulfate were purchased from Sigma-Aldrich (St. Louis, MO, USA). Phenolic acid standards (Vanillic, protocatechuic acid, Cinnamic acid, Chlorogenic, Gallic, Caffeic, and* p*-Coumaric), hydrochloric acid (37%) (HCl), and trolox were obtained from Sigma-Aldrich (Hamburg, Germany), while methanol, acetic acid, acetonitrile, and phosphoric acid used in the HPLC analyses were of HPLC grade and purchased from Thermo Fisher Scientific, (Loughborough, and Leicestershire, UK).

### 2.2. Collection of Plant Materials and Sample Preparation

Twenty kg of* C. nutans* was collected randomly from three different parts of the* C. nutans* garden at the YPL Herbal Farm, Jelebu, Seremban, Negeri Sembilan, Malaysia. Authentication was made by Dr. Shamsul Khamis at the Biodiversity unit of the Institute of Bioscience, Universiti Putra Malaysia, where the voucher specimen SK 2002/12 was deposited. The collected plant was washed thoroughly with running tap water and repeatedly with deionized water. The leaves and stem were individually freeze-dried (VirTis benchtop K, Bieleveld, Germany) and stored at −80°C prior to further analyses.

### 2.3. Solvent Extraction and Fractionation of Crude Extracts

**Figure 1:**
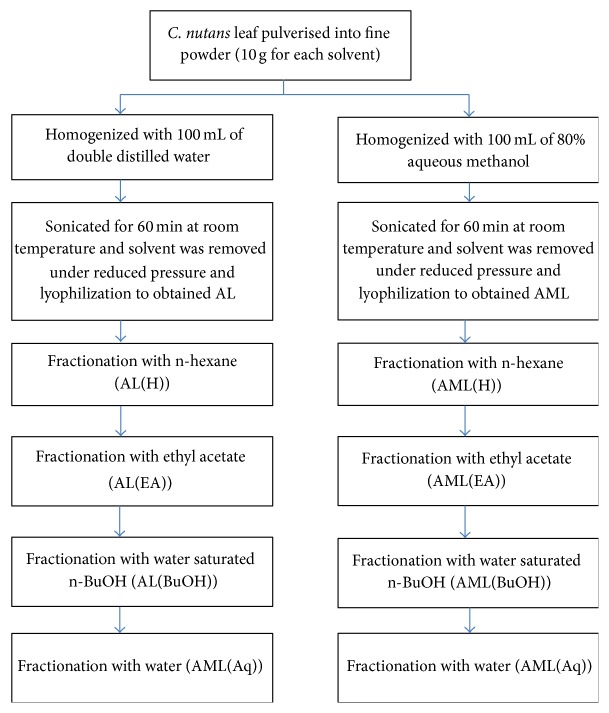
Flow chart showing the preparation of crude leaf extracts of* Clinacanthus nutans* (AL and AML), which were defatted using hexane (AL[H], AML[H]), ethyl acetate (AL[EA] and AML[EA]), and n-butanol (AL[BuOH] and AML[BuOH]), to obtain the final aqueous fractions (AL[Aq] and AML[Aq]).

The leaf and stem of* C. nutans* were, respectively, pulverised into fine powder using a stainless steel blender (Waring Commercial, Torrington, CT, USA) and passed through a mesh opening of 35 mm sieve. The leaf and stem were separately mixed with solvents of differing polarities: hexane, ethyl acetate, 80% methanol, water, and hot water (70°C) at the ratio of 1 : 10 (w/v). Then, these mixtures were sonicated for 60 min at 25°C in an ultrasonicator water bath (Power sonic 505, Hwa Shin Technology Co., Seoul, Korea). The mixtures were then individually filtered through Whatman filter paper No. 1 and the entire extraction process was repeated twice on the residue obtained from the previous filtration process. Subsequently, solvents were removed under reduced pressure (Rotavapor R210, Buchi, Flawil, Switzerland) followed by lyophilization (Virtis Benchtop K Freeze Dryer, SP Industries, Warminster, PA, USA). Following antioxidant activity tests (as detailed below), the hot water (AL) and 80% methanolic (AML) leaf extracts of* C. nutans* showed the best activities, and thus they were further fractionated as depicted in Figure 1. Briefly, the crude extracts (10 g of AML and AL each) were dispersed separately into 100 mL of double distilled water, and the solutions were partitioned with 100 mL of n-hexane by stirring for 60 min at room temperature to remove any residual lipids. Subsequently, the mixtures were left at room temperature until both solvent layers were well-separated from each other. After separation from the n-hexane layer, the aqueous layer was homogenized with 100 mL of ethyl acetate by magnetic stirrer for 60 min at room temperature. Consequently, the mixtures were left at room temperature until both solvent layers were well-separated from each other. Then, the aqueous layer after partitioning with n-hexane and ethyl acetate was homogenized with 100 mL of n-butanol. The partitioning procedure was repeated twice at each step and the solvents were pooled and concentrated to dryness under reduced pressure (Rotavapor R210, Buchi, Flawil, Switzerland). The remaining aqueous fraction (Aq) was subjected to lyophilization (Virtis Benchtop K Freeze Dryer, SP Industries, Warminster, PA, USA). The dried crude extracts and the fractions were stored at −80°C until further analysis.

### 2.4. Determination of Total Phenolic Contents (TPC) of Crude Extracts

The TPC were determined using Folin-Ciocalteu reagent as described by Ainsworth and Gillespie [17], and the results (*n* = 3) were expressed as milligram Gallic acid equivalent per gram extract (mg GAE/g extract).

### 2.5. Antioxidant Activity Assays of Crude Extracts and Fractions of the AL and AML Extracts

#### 2.5.1. DPPH Free Radical Scavenging Activity

The antioxidant activities of the extracts and fractions were determined using the DPPH radical assay according to the method described by Chan et al. [18], with Trolox as the standard. The radical scavenging activity of each extract was expressed as percentage of activity over the blank (*n* = 3).

#### 2.5.2. ABTS Radical Cation-Scavenging Activity

The ABTS free radical scavenging activity of each sample was determined as previously described by Iqbal et al. [19], and the results (*n* = 3) were expressed as milligram Trolox equivalents per gram extract (mg TE/g extract).

#### 2.5.3. Ferric Reducing Antioxidant Power (FRAP)

Total reducing capacity was determined by using ferricyanide method as described by Berker et al. [20], and the results (*n* = 3) were expressed as milligram Gallic acid equivalents in 1 gram extract (mg GAE/g extract).

### 2.6. Analysis of Selected Phenolic Compounds in Crude Extracts and Fractions of the AL and AML Extracts by HPLC-DAD

HPLC analysis was performed using Agilent G1310A pump linked with diode array detector (Agilent, Stevens Creek Blvd Santa Clara, USA). Chromatographic separations were performed on a LUNA C-18 column (5 mm, 250 × 4.6 mm) (Phenomenex, Torrance, CA, USA). The solvent composition and gradient elution conditions were described previously by Mariod et al. [21] with some modifications. The mobile phase was composed of solvent (A) water-acetic acid (94 : 6, v/v, pH 2.27) and solvent (B) acetonitrile. The solvent gradient was as follows: 0–15% B in 40 min, 15–45% B in 40 min, and 45–100% B in 10 min. A flow rate of 0.5 mL/min was used and 20 *µ*L of sample was injected. Samples and mobile phases were filtered through a 0.22 *μ*m Millipore filter, type GV (Millipore, Bedford, MA) prior to HPLC injection. Each extract and fraction were analyzed in three replicates. The standards used were protocatechuic acid, Ferulic acid, Gallic acid,* p*-Coumaric, Chlorogenic acid, Vanillic acid, and Caffeic acid and were measured at 320 nm. Phenolic compounds were identified and quantified by comparing their retention times with authenticated standards.

### 2.7. Animal Handling and Feeding

The high fat and high cholesterol (HFHC) diet was formulated according to Imam et al. [22], with minor modifications. Every kg of HFHC formulation contained 500 g ground standard rat chow, 25 g of cholesterol, 200 mL palm oil (instead of corn oil), 60 g fine sugar, 200 g Nespray full cream milk, and 50 g of starch (to cement the pellet together). The HFHC diet was dried in an oven at 60°C for 24 hours, cut into small equal-sized pieces, and fed to the rats to induce hypercholesterolemia.

Furthermore, healthy male Sprague-Dawley rats weighing about 200 g–250 g were housed in large stainless steel spacious cages, with free access to food and water. The animal house was ventilated with a 12-hour light/dark cycle at the ambient temperature of 25–30°C, throughout the experimental period. Rats were allowed to adapt to their environment for at least 10 days before the initiation of experiment. All experiments and protocols described in the study were approved by the Animal Ethics Committee (Project approval number: UPM/FPSK/PADS/BR-UUH/00484) of the Faculty of Medicine and Health Science, Universiti Putra Malaysia, Malaysia. The rats were randomly divided into nine groups of seven rats each; the normal control (NC) received normal pellet, while the control group received HFHC and the STATIN groups received HFHC + oral gavage of 10 mg/kg/day simvastatin. The aqueous leaf extract (AL) and aqueous methanolic leaf extract (AML) groups were given HFHC + oral gavage of 500, 250, or 125 mg/kg/day/rat of the respective extracts. At the end of the experimental period (49 days), the animals were fasted overnight and sacrificed by dissection method. The liver was excised immediately and washed with ice-cold saline prior to storage in RCL2 Solution (ALPHELYS, France) at −80°C. Blood was collected by cardiac puncture after an overnight fast and centrifuged at 3000 rpm for 10 min at 4°C to separate the serum.

### 2.8. Antioxidant Markers

#### 2.8.1. Serum Antioxidant Markers

Serum total antioxidant status (TAS), glutathione peroxidase (GPx), and SOD were analyzed using Randox analytical kits according to the manufacturer's instructions using Selectra XL instrument (Vita Scientiĕc, Dieren, Netherlands).

#### 2.8.2. Liver Electron Spin Resonance (ESR) Spectroscopy

The hepatic antioxidant capacities of rats against hydroxyl radical was measured using ESR spectrometer (Jeil FA100; Tokyo, Japan) as described by Imam et al. [22], and DMSO was used as standard.

### 2.9. Oxidative Stress Markers

#### 2.9.1. Thiobarbituric Acid Reactive Substances (TBARS)

TBARS was determined using the method described by Chan et al. [23], and MDA was used as the standard (*y* = 0.1982*x* − 0.1898, *R* ^2^ = 0.9947).

#### 2.9.2. *F*_2_-Isoprostane

Serum from blood collected in plain tubes was used for measurements of Serum *F*_2_-isoprostane using the respective ELISA kits according to the manufacturers' instructions. Absorbances were read on BioTeK Synergy H1 Hybrid Reader (BioTek Instruments Inc., Winooski, VT, USA) at the appropriate wavelengths (450 nm). The results were analyzed on http://www.myassays.com/ using four-parametric test curve; *F*_2_-isoprostane (*R* ^2^ = 1).

### 2.10. Hepatic mRNA Expression Level

Hepatic RNA was isolated using the Total RNA Isolation Kit (Vivantis, Malaysia) according to the kit protocol. Primers were designed on the GenomeLab eXpress Profiler software using* Rattus norvegicus* sequences adopted from the National Center for Biotechnology Information GenBank Database (http://www.ncbi.nlm.nih.gov/nucleotide/). The genes of interest, housekeeping genes, and an internal control are shown in Table 1. The forward and reverse primers had universal sequences (tags) in addition to nucleotides that were complementary to the target genes. Primers were supplied by First Base Ltd. (Selangor, Malaysia) and diluted in 1x Tris-EDTA buffer. Reverse transcription and multiplex PCR of RNA samples (50 ng each) were done in an XP Thermal Cycler (BIOER Technology, Hangzhou, China) according to the kit protocol, while PCR products (1 *μ*L each) were mixed with 38.5 *μ*L of sample loading solution and 0.5 *μ*L of DNA size standard 400 (Beckman Coulter, Inc., Miami, FL, USA) in a 96-well sample loading plate and analyzed in the GeXP machine (Beckman Coulter, Inc., Miami, FL, USA). The results were analyzed using the Fragment Analysis module of the GeXP system software and then imported into the analysis module of eXpress Profiler software. Normalization was done with GAPDH.

### 2.11. Statistical Analyses

Data were reported as mean ± standard deviation (*n* = 3 for antioxidant assays and 7 for animal study). Difference between each group was assessed by ANOVA accompanied by Duncan's multiple range test (SPSS for windows, version 17), and *p* < 0.05 was regarded as significant.

## 3. Results

### 3.1. Extraction Yield and TPC of Crude Extracts

The leaf and stem of* C. nutans* were extracted using solvents with differing polarities including hexane, ethyl acetate, 100% methanol, 80% methanol, hot water, and ambient water. The extraction yield increased with increasing solvent polarity, and the highest extraction yields were from the hot water leaf and stem extracts, while the lowest were the hexane leaf extract and ethyl acetate stem extract (Table 2). Generally, the leaf extracts showed higher TPC in comparison with the stem extracts (Table 2). The AML extract showed the highest TPC but was not significantly different from those of the water leaf extracts (HAL and AL), which were comparatively high (*p* > 0.05). A different trend was observed for the stem extracts, in which the EAS showed the highest TPC followed by the hot water extract (HAS), aqueous methanol extract (AMS), water extract (AS), 100% methanolic extract (MS), and hexane extract (HS).

### 3.2. Antioxidant Capacities and Phenolic Compositions of the Crude Leaf and Stem Extracts of* C. nutans*

**Figure 2:**
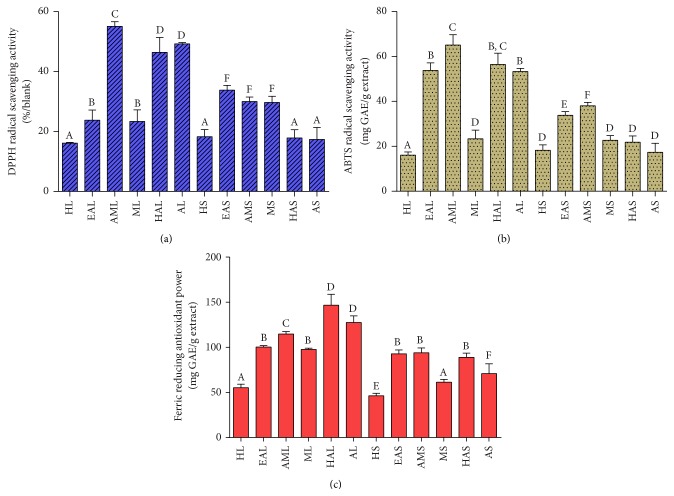
Antioxidant capacities of different solvent extracts of the leaf and stem of* Clinacanthus nutans* determined by (a) DPPH, (b) ABTS, and (c) FRAP Assays. Determinations for DPPH were expressed in percentage of radical scavenging activity over blank (%/blank), and those of ABTS assay were expressed as equivalent of Trolox, while, for FRAP assay, Gallic acid was used as calibration standard. Data are means of three replicates and are reported as mean ± standard deviation. Bars with different letters in each panel differ significantly (*p* < 0.05) according to Duncan's multiple range test. Groups are the same as Table 2.

As can be recalled, antioxidant activities were determined by DPPH, ABTS, and FRAP assays and expressed as mg GAE or TE/g extract. The estimated values of antioxidant activities by DPPH radical varied from 16.12 to 55.12% (Figure 2(a)). The highest DPPH radical scavenging activity was exhibited by the AML extract followed by the AL, HAL, EAS, AMS, MS, EAL, ML, HS, HL, HAS, and HS extracts. A similar trend was observed for the ABTS radical scavenging activities of the leaf and stem extracts. However, the EAL extract showed good ABTS radical scavenging activity almost comparable to those of the HAL and AL extracts, in contrast to the results of the DPPH radical scavenging activity (Figure 2(b)). A different trend was observed for FRAP, whereby the HAL extract showed the highest activity followed by those of the AL, AML, EAL, ML, and other stem extracts (Figure 2(c)).

**Figure 3:**
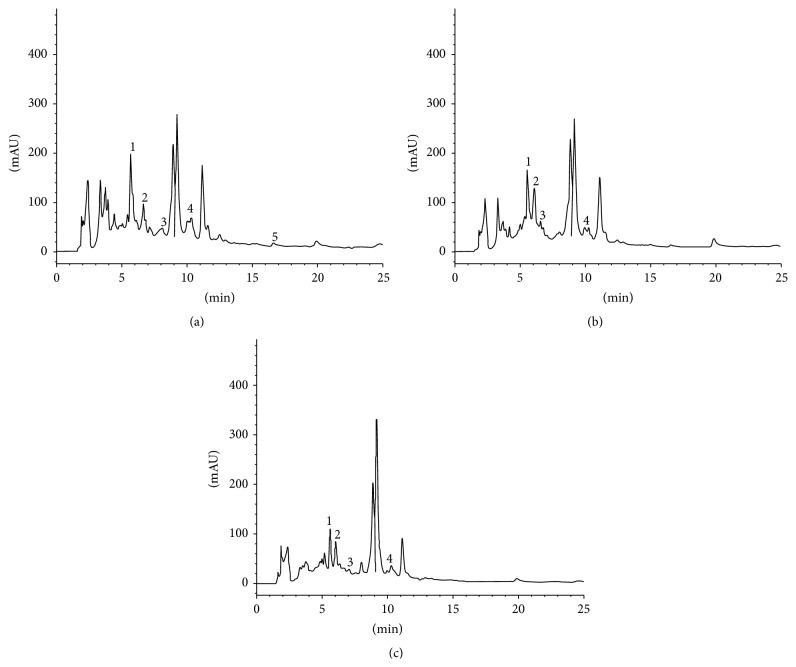
HPLC-DAD chromatogram of aqueous methanol leaf (a), aqueous leaf (b), and hot aqueous leaf (c) extract from* Clinacanthus nutans*. Peaks labelled with 1: protocatechuic acid, 2: Chlorogenic acid, 3: Caffeic acid, 4: Ferulic acid, and 5: Cinnamic acid.

The three most potent extracts (in terms of antioxidant capacities), which were the HAL, AL, and AML extracts, were subjected to phenolic acid composition analyses by HPLC-DAD (Figure 3, Table 3). Eight phenolic acids were tested, including Cinnamic acid, PCA, Vanillic acid, Gallic acid, Caffeic acid, Ferulic acid, Chlorogenic acid, and* p*-Coumaric acid. In all the three extracts, PCA was detected to be the major phenolic acid, followed by Chlorogenic acid and trace amounts of Ferulic acid and Caffeic acid. However,* p*-Coumaric acid, Vanillic acid, and Gallic acid were not detected in all the 3 tested extracts. Cinnamic acid was detected in trace amounts in the AML and HAL extracts, but not detected in the AL extract. Furthermore, the TPC detected by HLPC-DAD were 432.50, 409.54, and 398.54 mg/g extract for AML, HAL, and AL, respectively.

### 3.3. Antioxidant Capacities and Phenolic Compositions of the AML and AL Fractions

For practical reasons, the two extracts (AML and AL) with the highest TPC, PCA, and antioxidant capacities were chosen for fractionation. The yield and TPC contents of the AL and AML fractions are presented in Table 4. The TPC was found to be high in all the tested fractions, with the AL(EA) fraction being the highest followed by the AML(EA), AL(BuOH), AML, AML(BuOH), AL, and aqueous fractions (AL(Aq) and AML(Aq)), while the lowest were the AL(H) and AML(H) fractions, respectively (*p* < 0.05).

**Figure 4:**
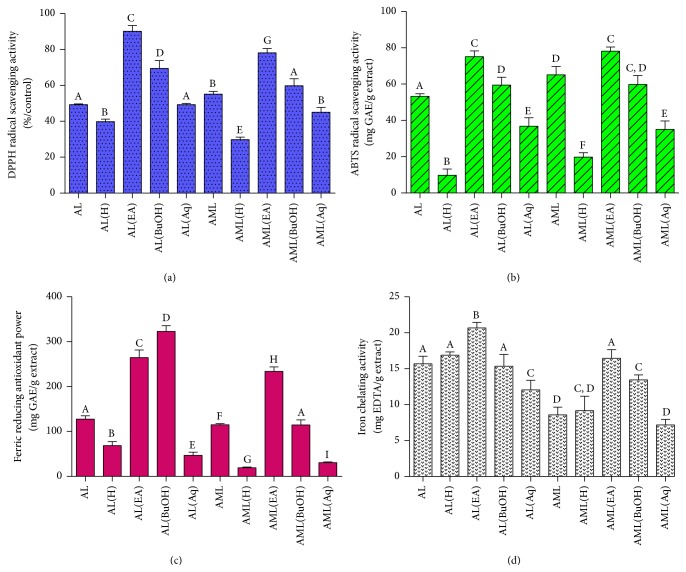
Antioxidant activities of crude leaf extracts and fractions of* C. nutans* determined by DPPH (a), ABTS (b), FRAP (c), and Iron Chelating (d). Determination for DPPH was expressed in percentage of radical scavenging activity over control (%/control), while, in ABTS and FRAP, Gallic acid and Trolox were used as calibration standards, respectively. Data are means of three replicates and data is reported as mean ± standard deviation. Bars with different letters (A–G) in each panel differ significantly (*p* < 0.05) according to Duncan's multiple range test. Groups are the same as Figure 1.

Furthermore, the antioxidant capacities of the AL and AML fractions of* C. nutans* were determined using multiple assays based on different mechanistic principles (Figure 4). Specifically, the DPPH radical scavenging activities of* C. nutans* extracts (Figure 4(a)) were in the order of EA > BuOH > Aq > crude (AL and AML) > hexane (*p* < 0.05). The EA fractions of both AL (90.13 ± 3.13%) and AML (78.12 ± 2.35%) showed significantly higher DPPH radical scavenging activities than other fractions (*p* < 0.05). Interestingly, both EA-AL and EA-AML showed 2-fold higher DPPH radical scavenging activities compared with the crude extracts (AL and AML), respectively (*p* < 0.05). Similar patterns were observed for the ABTS radical scavenging activities (Figure 4(b)), whereby the EA fractions showed the most radical scavenging capacity compared with the other fractions (*p* < 0.05). In this case, the ABTS radical scavenging activity of the AL-EA fraction was 7-fold greater than that of the AL extract, whereas that of the AML-EA fraction was 3-fold greater compared to that of the AML extract (*p* < 0.01). No significant differences were observed between the EA fractions of the AL and AML extracts (*p* > 0.05). Different trends were observed for the FRAP of the* C. nutans* extracts (Figure 4(c)), in which the AL-BuOH fraction showed the highest activity followed by the AL-EA, AML-EA, AML-BuOH, AL, AML, AL-H, AL-Aq, AML-Aq, and AML(H) fractions (*p* < 0.05).

Similarly, the phenolic composition of the AML and AL fractions of the AL and AML were analysed on HPLC (Table 5). HPLC-DAD detected PCA, Cinnamic acid, Gallic acid, Caffeic acid, Ferulic acid, and* p*-Coumaric acid in the AL fractions, in contrast with the AL crude extract which had PCA, Chlorogenic acid, Caffeic acid, and Ferulic acid. Also, the AML fractions had Vanillic acid, Gallic acid, and* p*-Coumaric acid, which were not in the AML crude extract. Interestingly, both crude extracts of* C. nutans* showed high amounts of PCA (33.28 ± 0.01 mg/g extract), while the PCA concentrations in both EA fractions were significantly concentrated at around 6-fold higher for AL-EA and 3.6-fold higher for AML-EA in comparison with the respective AL and AML crude extracts (*p* < 0.01).

Results from various antioxidant assays and phenolic composition showed that the most active fractions of the AL and AML crude extracts were the EA fractions, which had PCA contents concentrated up to 6-fold in comparison with crude extracts, thus the choice of the name PCA-rich fraction.

### 3.4. Animal Study

#### 3.4.1. *C. nutans* Extracts Slowed the Rate of Weight Gain Induced by HFHC Diet

**Figure 5:**
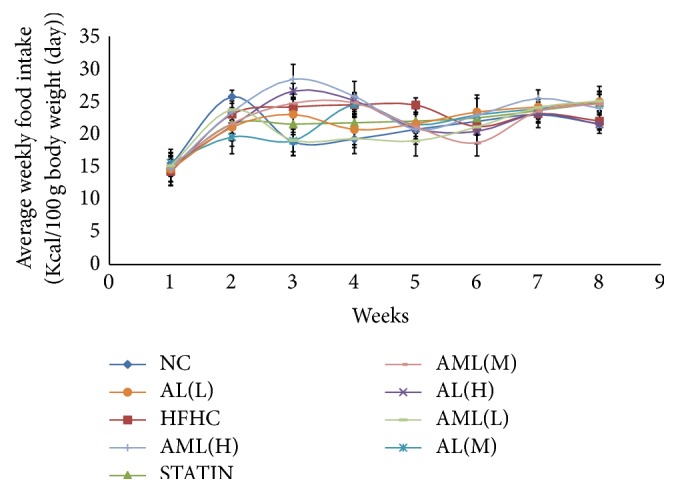
Average weekly food intake in high fat and high cholesterol fed rats after 7 weeks of intervention. Values are means ± SD (*n* = 7). NC: normal control group; HFHC: high fat and high cholesterol group; STATIN: HFHC + simvastatin (10 mg/kg/day/rat); AL(H): HFHC + high dose aqueous leaf extract (500 mg/kg/day/rat); AL(M): HFHC + medium dose aqueous leaf extract (250 mg/kg/day/rat); AL(L): HFHC + low dose aqueous leaf extract (125 mg/kg/day/rat); AML(H): HFHC + high dose aqueous methanol leaf extract (500 mg/kg/day/rat); AML(M): HFHC + medium dose aqueous methanol leaf extract (250 mg/kg/day/rat); AML(L): HFHC + low dose aqueous methanol leaf extract (125 mg/kg/day/rat).

**Figure 6:**
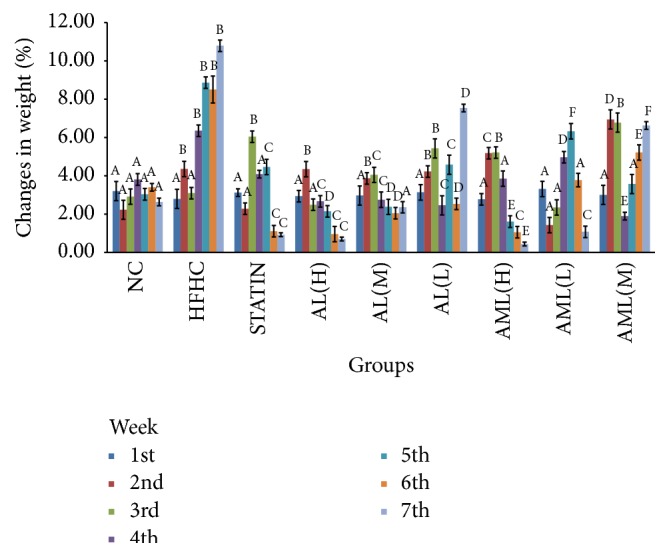
Weekly body weight changes in high fat and high cholesterol fed rats after 7 weeks of intervention. Values are means ± SD (*n* = 7). Bars representing weekly mean weights labelled with different letters (A–F) are significantly different between the groups (*p* < 0.05) according to Duncan's multiple range test. Groups are the same as Figure 5.

Figure 5 shows the caloric intakes of the different animal groups throughout the experimental period. Food consumption (g/100 g/day) was significantly lower (*p* < 0.05) in the groups that received HFHC compared with the NC group, although the total calories consumed were the same for all groups. Weight changes are depicted in Figure 6, which showed progressive weight gain in all groups with time. In particular, the HFHC group showed significant increase in body weight gain throughout the intervention period, in comparison with the AL- and AML-treated experimental rats, which had dose-dependent reductions in weight gain (*p* < 0.05). Interestingly, at the end of the intervention period, the STATIN, AL(H), and AML(H) groups had the least weight gains (*p* < 0.05).

#### 3.4.2. Lipid Profile Analysis

The lipid profiles obtained at the end of the intervention are shown in Table 6. From the results, it was observed that the HFHC caused significant elevation of the TC level by 202% compared with the NC group. On the other hand, there was significant reduction of TC by 37%, 28%, and 18% in the STATIN, AML(H), and AL(H) groups, respectively, compared with the HFHC group. Furthermore, there was a 3-fold decrease in HDL of the HFHC group compared with the NC group. On the other hand, the treated groups showed significantly higher HDL levels compared with the HFHC group except the AL(M) and AL(L). The LDL levels were similarly elevated in the HFHC group by 184% compared with the NC group, and there were dose-dependent decreases in the AL groups, while the AML groups did not show such differences. The serum VLDL of the HFHC group was elevated by 92% compared with the NC group. The treated groups showed significant reductions in VLDL levels compared with the HFHC group, except for the AL(L) group. TG levels in the HFHC group were slightly higher than in the treated groups, although only the AML(H) had significantly lower level (*p* < 0.05), which was comparable to that of the NC group.

#### 3.4.3. Serum and Hepatic Markers of Antioxidant Status

**Figure 7:**
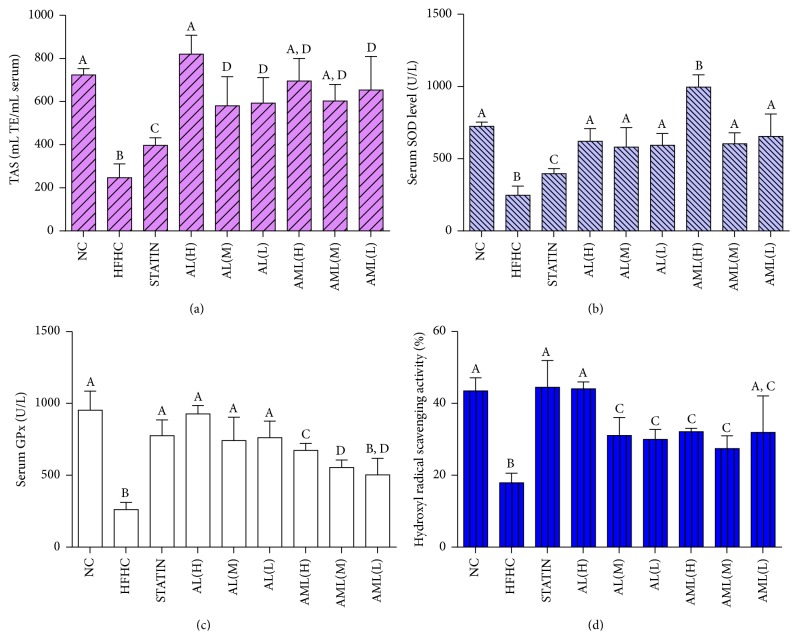
Effects of* Clinacanthus nutans* extracts on (a) serum total antioxidant status (TAS), (b) serum superoxide dismutase (SOD), (c) serum glutathione peroxidase (GPx), and (d) liver hydroxyl radical scavenging activity, in high fat and high cholesterol fed rats after 7 weeks of intervention. Bars and error bars represent mean ± standard deviation (*n* = 7). Bars representing different groups in each panel with different letters are significantly different (*p* < 0.05). Groups are the same as Figure 5.

The consumption of the HFHC diet significantly reduced antioxidant capacity based on the serum TAS, SOD and GPx, and hepatic OH radical scavenging activity in comparison with the control group (*p* < 0.05) (Figure 7). The serum total antioxidant level was significantly elevated for the all the treated groups. Interestingly, antioxidant capacities for the high dose groups of the AL and AML extracts were as high as that of the NC group. There was a significant inhibition of the serum antioxidant status in the HFHC group, as shown by the reduced SOD activity, which was significantly enhanced in groups treated with* C. nutans* (*p* < 0.05) and higher than that of the simvastatin-treated group (*p* < 0.05). Similarly, the serum GPx activity was elevated in the* C. nutans*-treated groups compared with the HFHC group (except the AML(L) group). Additionally, the hepatic hydroxyl radical scavenging activity was evaluated, which indicated that long term HFHC diet reduced the scavenging activity by 2-fold in comparison with the NC group.* C. nutans*-treated groups showed significantly elevated hepatic hydroxyl radical scavenging activities especially in the AL(H) group.

#### 3.4.4. Serum and Hepatic Markers of Oxidative Stress

**Figure 8:**
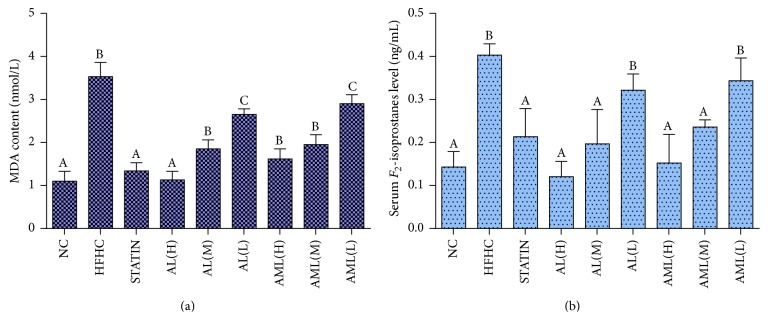
Effects of* Clinacanthus nutans* extracts on (a) hepatic MDA content, (b) serum *F*_2_-isoprostanes level in high fat and high cholesterol fed rats after 7 weeks of intervention. Bars and error bars represent mean ± standard deviation (*n* = 7). Bars representing different groups in each panel with different letters are significantly different (*p* < 0.05). Groups are the same as Figure 5.

The extent of lipid peroxidation in the liver was determined using the MDA content. This method was also used to evaluate the redox equilibrium in the rats fed with the HFHC diets. Hepatic MDA content in the HFHC group was elevated by 3.5-fold in comparison with the NC group (Figure 8(a)). All the treated groups showed significantly lower levels of hepatic MDA, dose-dependently, compared with the HFHC group (*p* < 0.05). Serum lipid peroxidation was determined by *F*_2_-isoprostanes (Figure 8(b)). Figure 8(b) shows that the serum *F*_2_-isoprostanes levels were increased approximately 4-fold in the HFHC group, while the treated groups showed significantly lower levels of *F*_2_-isoprostanes except the AL(L) and AML(L) groups. The AL(H) group had the lowest level of *F*_2_-isoprostanes.

#### 3.4.5. Effects of* C. nutans* Crude Extracts on mRNA Levels of Antioxidant Genes

**Figure 9:**
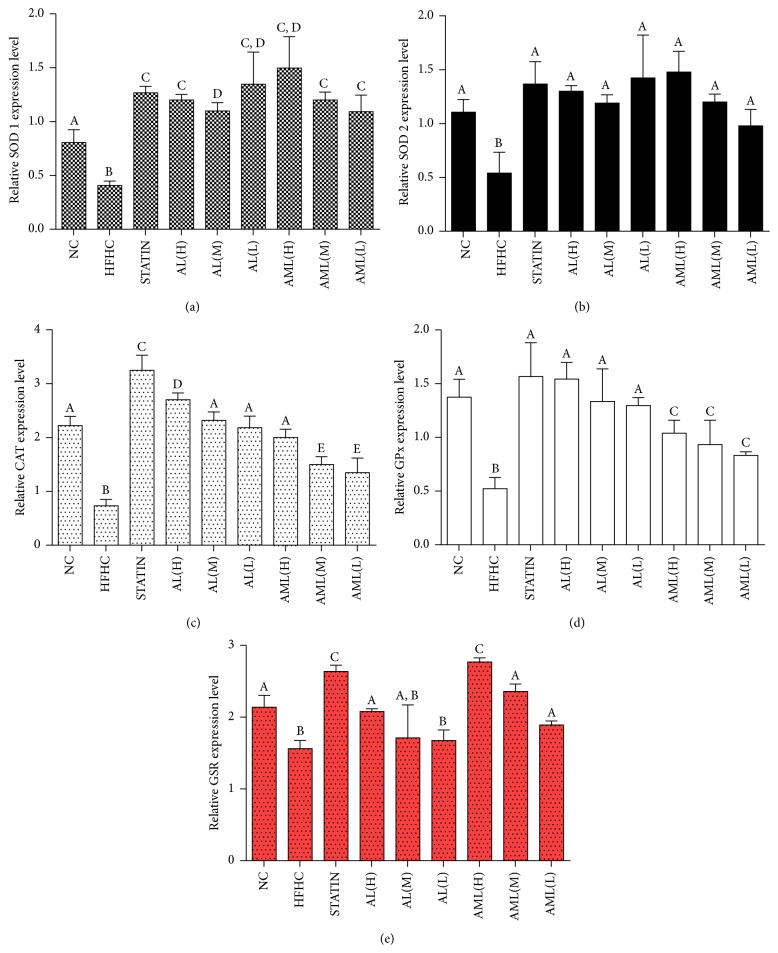
Effects of* Clinacanthus nutans* extracts on the mRNA levels of hepatic antioxidant genes in high fat and high cholesterol fed rats after 7 weeks of intervention. Bars and error bars represent mean ± standard deviation (*n* = 7). Bars representing different groups in each panel with different letters are significantly different (*p* < 0.05). CAT: catalase; GPx: glutathione peroxidase; GSR: glutathione peroxidase; SOD: superoxide dismutase. Groups are the same as Figure 5.

The mRNA levels of hepatic antioxidant genes (SOD 1, SOD 2, CAT, G-Px, and GSR) were studied using Multiplex GeXP genetic analysis system, with KanR as the internal control. As shown in Figure 9, the AL and AML groups upregulated the expression of the antioxidant genes in comparison to the HFHC and NC groups. There was significant upregulation of the SOD 1 gene in the treated rats compared to the HFHC group (*p* < 0.05). Similarly, SOD 2 was upregulated in the treated groups although not different from the HFHC group except for the statin group (*p* > 0.05). The mRNA levels of CAT in the AML and AL groups were found to be higher than in the HFHC group, in a dose-dependent manner. Interestingly, the statin group showed a significantly higher level compared to the other groups. The expression levels of GPx in the AL-treated groups were significantly upregulated up to the level of the NC group. However, for the AML-treated group, even though the expression levels showed increases in a dose-dependent manner, no significant differences were observed among the different dose-treated groups (*p* > 0.05). A different trend was observed in the expression levels of GSR, whereby the AML groups showed upregulation in a dose-dependent manner, but only the AL(H) group was significantly different compared to the HFHC group.

## 4. Discussion

As can be recalled, based on the TPC results and those of other antioxidant assays (DPPH, ABTS, and FRAP), the AML, AL, and HAL extracts had the most antioxidant potentials, hence their detailed compositional analyses and subsequent use for the animal study. Moreover, numerous findings have shown that high phenolic content correlates with good antioxidant capacity and better attenuation of oxidative stress-related chronic diseases [5, 24]. Based on the strong antioxidant potentials, we hypothesized that the extracts would have potent antioxidative effects. Moreover, the phenolic compounds detected in the three most active extracts (HAL, AL, and AML), PCA, Chlorogenic acid, Cinnamic acid, Ferulic acid, Vanillic acid, Gallic acid, and Caffeic acid have all been reported to possess potent antioxidant properties. Accordingly, PCA has been demonstrated to prevent oxidative stress-related diseases such as CVD and cancer [25, 26]. Chlorogenic acid is also known to have potent antioxidant effects, which may explain the superior DPPH and ABTS radical scavenging of the AML extract compared with the HAL and AL extracts [27]. Additionally, Cinnamic acid may have contributed to the antioxidant properties of the HAL and AML extracts [28], while Vanillic acid, which was only detected in the AL extract, may have only contributed minimally to the antioxidant activity of the extract. Moreover, Prince et al. [29] had demonstrated that Vanillic acid is a potent antioxidant capable of protecting against lipid peroxidation. In general, however, based on the presence of multiple phenolics in the extracts of* C. nutans*, it is likely that synergism played a role in their overall bioactivities. The presence of PCA as the major phenolic in the EA fractions suggested that it may have contributed significantly to the bioactivity of the* C. nutans* extracts since the A fractions also showed potent antioxidant effects. Increased release of bound phenolics during partitioning may have also contributed to the high amounts of phenolics detected in the fractions.

High fat diet feeding can cause disorders like hyperlipidemia, insulin resistance, and CVD, which resemble the human metabolic syndrome closely [30–33]. The present study demonstrated that rats fed a HFHC diet showed approximately 200%, 79%, and 95% higher concentrations of serum TC, LDL, and TG, respectively, and 36% lower HDL compared with rats fed a normal rat chow diet (*p* < 0.05), confirming hyperlipidemic condition (Table 6). Additionally,* C. nutans* supplementation in the rats showed evidence of attenuating hyperlipidemia-associated oxidative stress [34]. Moreover, there were improvements in antioxidant enzymes activities in serum (Figure 7) with underlying transcriptional changes in the antioxidant genes (Figure 9), which may have been the basis for the improved antioxidant capacities of the serum and liver of the rats. Furthermore, we observed significant increases in oxidative stress markers (MDA and *F*_2_ isoprostane) in the HFHC group (Figure 8), in keeping with the reduced antioxidant status due to hyperlipidemia-induced oxidative stress [5, 34]. Conversely, administration of the phenolic-rich extracts from* C. nutans* significantly decreased the hyperlipidemia-induced oxidative stress markers in rats. The attenuation of lipid peroxidation in the* C. nutans*-treated animals, especially at the higher dose, suggested that the rats had reduced risk of cardiometabolic diseases, since lipid peroxidation is known to promote the diseases [35]. Moreover, *F*_2_-isoprostanes which are clinically relevant and potent indicators of oxidative stress [36] were attenuated by* C. nutans* supplementation. The ability of the* C. nutans* extracts to attenuate hyperlipidemia-induced oxidative stress is indicative of their rich phenolic contents. Moreover, phenolics such as PCA, Chlorogenic acid, and Caffeic acid have been shown to reduce biomarkers of oxidative stress like MDA and *F*_2_-isoprostanes and increase the activities of antioxidant enzymes like CAT, SOD, GPx, or Gsr [7, 9].

The use of single, mostly synthetic, antioxidant compounds has not proved effective in reducing overall chronic disease burden and mortality likely due to the limited effects on metabolic processes or the metabolic compensation from other systems in the presence of the single antioxidants [37]. This has generated interests in bioactive-rich fractions, which have been demonstrated to be more effective than single compounds, and can potentially have effects across different metabolic pathways, thus reducing the chances of any metabolic compensatory process canceling out the effects of the antioxidants in the rich-fraction [16]. In this study, the different phenolic compounds in the extracts may have acted through different mechanisms including the scavenging of free radicals, electron transfer, and neutralization of the free radicals to attenuate the hyperlipidemia-induced oxidative stress. These may have prevented the oxidation of biomolecules, as suggested by the reduced oxidative stress products in the* C. nutans*-treated groups in this study, in addition to the regulation of cholesterol levels possibly through modulating its metabolism. Similarly, the phenolic compounds in the extracts may have regulated the c-Jun-N-terminal kinase, thereby modulating oxygen radical production and inflammation [3, 6].

## 5. Conclusions

The present study demonstrated the antioxidant capacities of* C. nutans* extracts and their efficacies against hypercholesterolemia-induced oxidative stress in rats. The results indicated that* C. nutans* is rich in multiple natural antioxidants, and hence its effects may be contributed by multiple bioactive compounds. Thus,* C. nutans* may be a good source of functional ingredients that can be used for managing oxidative stress-related diseases. However, future clinical studies including detailed toxicity analyses are needed to determine the usefulness of this plant in managing such diseases.

## Supplementary Material

The table shows the food composition of the normal pellet, and High Fat and High Cholesterol (HFHC) Diets. The rats were allowed to adapt to their environment for at least 10 days on normal pellet diet prior to commencement of interventions. The rats were then randomly divided into nine groups of seven rats each; the normal control (NC) received normal pellet, while the other groups received HFHC to induce hypercholesterolemia in addition to the respective treatments. Every kg of the HFHC formulation contained higher cholesterol, fats and protein compared to the normal pellet.

## Figures and Tables

**Table 1:** Gene name, accession number, and sequences of primers used in multiplex panel analysis.

Gene name		Primer sequence (with universal tag)	
		Forward primer	Reverse primer
CAT	NM_012520	AGGTGACACTATAGAATACATTCTATACGAAGGTGTTG	GTACGACTCACTATAGGGAGGTGTGAATTGCATTCTTAG
SOD1	NM_017050	AGGTGACACTATAGAATATCAATATGGGGACAATACAC	GTACGACTCACTATAGGGATACTTTCTTCATTTCCACCTT
SOD2	NM_017051	AGGTGACACTATAGAATAACTTTGGGTCTTTTGAGAA	GTACGACTCACTATAGGGATTCACTTCTTGCAAACTATG
GPx1	NM_030826	AGGTGACACTATAGAATAGGCAAGAATGAAGAGATTC	GTACGACTCACTATAGGGACTACCAGGAACTTCTCAAAG
GSR	NM_053906	AGGTGACACTATAGAATAAGCCTGGGGATAACCAGTGA	GTACGACTCACTATAGGGAAATGTAACCGGCACCCACAA
PPIA^a^	NM_017101	AGGTGACACTATAGAATATTCTGTAGCTCAGGAGAGCA	GTACGACTCACTATAGGGATTGAAGGGGAATGAGGAAAA
GAPDH^a,*∗*^	NM_017008	AGGTGACACTATAGAATAATGACTCTACCCACGGCAAG	GTACGACTCACTATAGGGAAGCATCACCCCATTTGATGT
KanR^b^			

^a^Housekeeping gene. ^b^Internal control. ^*∗*^Normalization gene. Reverse transcription (RT) and PCR were done according to manufacturer's instructions; RT reaction was at 48°C for 1 min; 37°C for 5 min; 42°C for 60 min; 95°C for 5 min and then held at 4°C, while PCR was as follows: initial denaturation at 95°C for 10 min, followed by two-step cycles of 94°C for 30 sec and 55°C for 30 sec, ending in a single-extension cycle of 68°C for 1 min. CAT: catalase; GAPDH: glyceraldehyde 3-phosphate dehydrogenase; GPx: glutathione peroxidase; GSR: glutathione reductase; KanR: kanamycin resistant; PPIA: cyclophilin A; SOD: superoxide dismutase.

**Table 2:** Extraction yield and total phenolic contents (TPC) of the crude leaf and stem extracts of *Clinacanthus nutans*.

Sample extracts	Extraction yield	TPC
	(g/100 g sample)	(mg GAE/g extract)
HL	3.07 ± 0.14^a^	27.60 ± 3.88^a^
EAL	3.96 ± 0.97^a^	50.14 ± 1.69^b^
AML	19.6 ± 0.77^b^	73.33 ± 12.18^c^
ML	12.24 ± 0.74^c^	48.84 ± 1.33^b^
HAL	22.50 ± 1.85^b^	69.73 ± 3.69^c^
AL	20.51 ± 0.14^b^	63.77 ± 7.31^c^
HS	2.04 ± 0.16^d^	23.15 ± 2.78^a^
EAS	1.00 ± 0.07^e^	52.91 ± 0.27^b^
AMS	12.06 ± 0.97^c^	40.91 ± 0.17^c^
MS	11.45 ± 0.74^c^	30.67 ± 3.07^d^
HAS	22.30 ± 1.85^b^	44.48 ± 4.64^e^
AS	10.67 ± 0.14^c^	35.45 ± 10.93^a,c,d,e^

Data for extraction yield and TPC are means of three replicates and data are reported as mean ± standard deviation (*n* = 3). Means within each column with different letters differ significantly (*p* < 0.05) according to Duncan's multiple range tests.

HL: hexane leaf extract; EAL: ethyl acetate leaf extract; AML: aqueous methanol leaf extract; ML: 100% methanol extract; HAL: hot aqueous leaf extract; AL: aqueous leaf extract; HS: hexane stem extract; EAS: ethyl acetate stem extract; AMS: aqueous methanol stem extract; MS: 100% methanol stem extract; HAS: hot aqueous stem extract; AS: aqueous stem extract.

**Table 3:** Phenolic composition of the extracts from the leaf of *Clinacanthus nutans*.

Phenolic compound	Individual phenolic content in *C*. *nutans* extracts (mg/g extract)		
	Aqueous methanol leaf	Hot aqueous leaf	Aqueous leaf
	(AML)	(HAL)	(AL)
Cinnamic acid	0.64 ± 0.01^a^	1.00 ± 0.02^a^	ND
Protocatechuic acid	33.28 ± 0.01^b^	33.28 ± 0.12^b^	33.28 ± 0.12^a^
Caffeic acid	3.62 ± 0.04^c^	5.32 ± 0.09^c^	5.11 ± 0.04^b^
Ferulic acid	1.33 ± 0.02^d^	10.39 ± 0.58^d^	1.49 ± 0.06^c^
Chlorogenic acid	21.38 ± 0.61^e^	25.24 ± 5.14^b^	22.84 ± 9.14^d^
Total phenolic	60.25 ± 0.69	75.23 ± 0.58	62.72 ± 9.26

Data of phenolic compositions are means of three replicates and the data is reported as mean (*n* = 3) ± standard deviation. Means within each column labelled with different letters are significantly different (*p* < 0.05) according to Duncan's multiple range test. ND: non detected. Groups are the same as Table 2.

**Table 4:** Extraction yield and total phenolic contents (TPC) of the AL and AML fractions.

Sample fractions	Extraction yield	Total phenolic content (TPC)
	(g/100 g sample)	(mg GAE/g extract)
AL	20.51 ± 0.14^a^	63.77 ± 7.31^a^
AL(H)	2.04 ± 0.16^b^	13.15 ± 2.78^b^
AL(EA)	1.00 ± 0.37^c^	128.83 ± 0.58^c^
AL(BuOH)	6.06 ± 0.97^d^	80.91 ± 0.49^d^
AL(Aq)	10.45 ± 0.74^e^	38.67 ± 4.07^e^
AML	19.60 ± 0.77^a^	73.33 ± 12.18^f^
AML(H)	2.07 ± 0.14^b^	27.60 ± 3.88^g^
AML(EA)	1.36 ± 0.97^c^	120.14 ± 1.69^h^
AML(BuOH)	3.06 ± 0.80^e^	66.34 ± 3.69^i^
AML(Aq)	12.24 ± 0.74^f^	48.84 ± 1.33^j^

Data of extraction yield and TPC is means of three replicates and data is reported as mean ± standard deviation. Means within each column with different letters differ significantly (*p* < 0.05) according to Duncan's multiple range tests. Groups are the same as Figure 1.

**Table 5:** Phenolic composition of the crude leaf extracts and fractions of *Clinacanthus nutans*.

Extract	Individual Phenolic Content in *C*. *nutans* extracts (mg/g extract)								
	Cinnamic acid	Protocatechuic	Vanillic acid	Gallic acid	Caffeic acid	Ferulic acid	Chlorogenic acid	*p-*Coumaric acid	Total phenolic content
AL	ND	33.28 ± 0.32^a^	ND	ND	5.11 ± 0.04^a^	1.49 ± 0.06^a^	22.84 ± 9.14^a^	ND	62.68 ± 9.26^a^
AL(EA)	2.12 ± 0.03^a^	198.06 ± 0.62^b^	ND	4.50 ± 0.05^a^	ND	33.09 ± 0.26^b^	ND	39.80 ± 0.21^a^	278.00 ± 1.18^b^
AL(BuOH)	2.64 ± 0.02^a^	66.17 ± 1.23^c^	ND	11.20 ± 0.01^b^	9.72 ± 0.51^e^	22.94 ± 0.81^c^	ND	43.23 ± 0.59^d^	155.90 ± 4.35^c^
AL(Aq)	ND	13.70 ± 1.56^d^	ND	2.49 ± 0.02^c^	ND	0.44 ± 0.03^d^	ND	0.96 ± 0.04^b^	17.59 ± 1.65^d^
AML	0.64 ± 0.01^c^	33.28 ± 0.01^a^	ND	ND	3.62 ± 0.04^c^	1.33 ± 0.02^e^	21.38 ± 0.61^b^	ND	60.25 ± 0.69^e^
AML(EA)	6.12 ± 0.03^d^	122.23 ± 1.24^e^	42.90 ± 0.17	16.49 ± 0.28^d^	6.53 ± 0.04^d^	25.16 ± 0.09^f^	15.44 ± 0.12^d^	12.72 ± 0.24^c^	247.59 ± 2.21^f^
AML(BuOH)	3.74 ± 0.26^e^	46.83 ± 1.10^f^	ND	12.03 ± 0.11^e^	ND	35.16 ± 0.07^g^	31.00 ± 0.81^e^	32.93 ± 0.07^d^	161.69 ± 2.42^g^
AML(Aq)	ND	17.77 ± 10.02^g^	ND	ND	4.28 ± 0.04^e^	0.62 ± 0.02^h^	40.28 ± 0.03^f^	ND	62.95 ± 10.11^e^

Data of phenolic compositions are means of three replicates and data is reported as mean (*n* = 3) ± standard deviation (*n* = 3). Means within each column labelled with different letters are significantly (*p* < 0.05) different according to Duncan's multiple range test. ND: nondetected. Groups are the same as Figure 1.

**Table 6:** Effect of *Clinacanthus nutans* on lipid profile in high fat and high cholesterol fed rats after 7 weeks of intervention.

Group	Lipid profile (mmol/L)				
	TC	HDL	LDL	VLDL	TG
NC	1.06 ± 0.14^a^	0.33 ± 0.02^a^	1.15 ± 0.31^a^	0.14 ± 0.01^a^	0.30 ± 0.03^a^
HFHC	3.21 ± 0.2^b^	0.14 ± 0.01^b^	3.27 ± 0.08^b^	0.27 ± 0.04^b^	0.49 ± 0.07^b^
STATIN	2.01 ± 0.10^c^	0.29 ± 0.05^a^	1.91 ± 0.18^c^	0.17 ± 0.05^a^	0.37 ± 0.10^a,b^
AL(H)	2.62 ± 0.34^d^	0.20 ± 0.03^c^	2.37 ± 0.22^d^	0.18 ± 0.04^a^	0.39 ± 0.10^a,b^
AL(M)	2.84 ± 0.09^d^	0.20 ± 0.07^b,c^	2.83 ± 0.19^e^	0.19 ± 0.01^c^	0.42 ± 0.03^b^
AL(L)	3.04 ± 0.27^b,d^	0.16 ± 0.06^b,c^	3.18 ± 0.57^b,e^	0.19 ± 0.05^a,b,c^	0.43 ± 0.10^a,b^
AML(H)	2.31 ± 0.24^c,d^	0.27 ± 0.09^a,c^	2.44 ± 0.31^d,e^	0.12 ± 0.03^a,c^	0.33 ± 0.02^a^
AML(M)	2.62 ± 0.06^d^	0.22 ± 0.03^c^	2.63 ± 0.41^d,e^	0.19 ± 0.03^c^	0.43 ± 0.07^b^
AML(L)	2.77 ± 0.17^d^	0.21 ± 0.01^c^	2.56 ± 0.32^d,e^	0.21 ± 0.01^c^	0.46 ± 0.03^b^

Values were means ± SD (*n* = 7). Means within each column labelled with different letters (a–c) are significantly (*p* < 0.05) different according to Duncan's multiple range test. Groups are the same as Figure 5.

## Abbreviations

AL: Aqueous leaf
AML: Aqueous methanol leaf
HFHC: High fat and high cholesterol
CVD: Cardiovascular disease
PCA: Protocatechuic acid
FRAP: Ferric reducing antioxidant power
TPC: Total phenolic content
HPLC: High performance liquid chromatography.
