# Supplementary material for: Phenolic Rich Extract from Clinacanthus nutans Attenuates Hyperlipidemia-Associated Oxidative Stress in Rats
Source: Oxid Med Cell Longev. 2016 Jan 10;2016:4137908. doi: 10.1155/2016/4137908 (PMC4737004; doi:10.1155/2016/4137908)
Supplement: Supplementary file 1 — The table shows the food composition of the normal pellet, and High Fat and High Cholesterol (HFHC) Diets. The rats were allowed to adapt to their environment for at least 10 days on normal pellet diet prior to commencement of interventions. The rats were then randomly divided into nine groups of seven rats each; the normal control (NC) received normal pellet, while the other groups received HFHC to induce hypercholesterolemia in addition to the respective treatments. Every kg of the HFHC formulation contained higher cholesterol, fats and protein compared to the normal pellet. [file 4137908.f1.pdf]

Supplementary data

Food Composition of the Normal Pellet and High Fat and High Cholesterol (HFHC) Diet

Food classes	Normal Pellet (%)	HFHC (%)
Carbohydrates	60.0	40.0
Protein	25.0	30.0
Vitamin mix	5.0	2.5
Fats	5.0	22.5
Fibre	5.0	2.5
Cholesterol	0.0	2.5
